# Self-Reported Beneficial Effects of Chinese Calligraphy Handwriting Training for Individuals with Mild Cognitive Impairment: An Exploratory Study

**DOI:** 10.3390/ijerph20021031

**Published:** 2023-01-06

**Authors:** Chih-Chun Hsiao, Chun-Chieh Lin, Chun-Gu Cheng, Yin-Han Chang, Hui-Chen Lin, Hsing-Chen Wu, Chun-An Cheng

**Affiliations:** 1 Department of Nursing, Taoyuan Armed Forces General Hospital, Taoyuan 32549, Taiwan; 2 Department of Neurology, Tri-Service General Hospital, National Defense Medical Center, Taipei 11490, Taiwan; 3 Department of Emergency Medicine, Taoyuan Armed Forces General Hospital, National Defense Medical Center, Taoyuan 32549, Taiwan; 4 Department of Emergency Medicine, Tri-Service General Hospital, National Defense Medical Center, Taipei 11490, Taiwan; 5 Department of Emergency and Critical Medicine, Wan Fang Hospital, Taipei Medical University, Taipei 11696, Taiwan; 6 Department of Psychology, National Taiwan University, Taipei 10621, Taiwan; 7 School of Nursing, Taipei Medical University, Taipei 11031, Taiwan; 8 Graduate Institute of Vocational and Technological Education, National Pingtung University of Science and Technology, Pingtung 91201, Taiwan

**Keywords:** Chinese calligraphy handwriting, mild cognitive impairment, benefit

## Abstract

Background: Dementia is a common disease in aging populations. The treatment has mainly focused on memory decline prevention and behavior control. Nonpharmacological treatments, such as cognition training, physical exercise, and music therapy have been effective in slowing memory decline. Chinese calligraphy handwriting (CCH) through breath regulation and fine hand control involves high concentration levels, emotion regulation, and self-awareness. CCH is a mind and body activity that is culturally relevant to older Chinese adults. This study evaluated the beneficial effects of CCH on mild cognitive impairment. Methods: In 2018, we conducted 8 weeks of CCH training at the Tri-Service General Hospital. The participants were asked to copy a regular script. At the end of the course, they gave oral presentations and showed their work. Self-report questionnaires on emotion, memory, upper limb coordination, attention, and language were collected before and after training. Results: The five questionnaires showed significantly positive feelings after CCH training. The conditions of emotional stability, concentration, hand movement, memory, and speech improved. Conclusions: CCH training stimulated the brain and improved cognition, psychological symptoms, and hand stability. It is inexpensive and worthwhile for elderly Chinese individuals with mild cognitive impairment to take time daily to practice calligraphy.

## 1. Introduction

Dementia is a progressive neurodegenerative disease that mainly manifests as memory and cognitive deterioration, dysfunction in activities of daily living, and neuropsychological and behavioral abnormalities [1]. Studies have shown that older patients have a higher incidence of dementia. According to the International Dementia Association Global Dementia Report, it was estimated that there are more than 50 million people with dementia worldwide, and this population is expected to grow to 152 million by 2050 [2]. Based on these statistics, one-thirteenth of the population over age 65 years old has dementia and one-fifth of individuals over age 80 have dementia [3]. Individuals with dementia are treated with acetylcholinesterase inhibitors and glutamate regulators to prevent memory decline, but there is no cure for dementia [4]. It is necessary to incorporate nondrug therapies to reduce mental, behavioral, and apraxia symptoms.

Nondrug therapies to slow disease progression have included cognitive training, reminiscence therapy, art therapy, music therapy, horticultural therapy, and massage [5]. Chinese calligraphy handwriting (CCH) is a unique form of nondrug therapy for Chinese patients. CCH is taught from elementary school to senior high school in Taiwan for purposes of hand-eye coordination and concentration. CCH performance integrates the mind and body and involves visual perception, spatial structures, and planning. The basic structure of Chinese characters is divided into form, sound, and meaning; the writer needs to understand the structure and the corresponding strokes. Before writing, participants must focus on the audiovisual stimuli of Chinese words then control any aspects of the breath and screen out miscellaneous worries to concentrate on their brush strokes [6,7]. The practice of CCH involves clearing all clutter, relaxing the body and sitting up straight, holding the upper arm at a precise angle, lifting the calligraphy pen, lightly lifting with an inhalation, and pressing the pen to the paper with an exhalation while moving through the order of brush strokes step by step. The right spirit with breath tone regulation influences the finished strokes. CCH can have physiological and psychological effects [8]. It has been shown that respiration decelerates, heart rate decreases, blood pressure decreases, and muscle tension relaxes with CCH training [9]. Evidence suggests that CCH training provides meaning in addition to mental exercises.

Taiwan formally became a hyperaged society in April 2018. Regarding age-related diseases, cognitive function degradation is common among older individuals. CCH has been suggested as a means of slowing cognitive decline. Elderly individuals have been encouraged to practice calligraphy frequently and to think about the older hieroglyphs and their equivalent modern characters, which enhances brain activity with visual, motor, and language stimulation. A previous study found that CCH improved memory and emotional and motor functions by objective evaluation [10]. This study evaluated the relationship between CCH and an enriched environment and health preservation by surveying participants.

The aims of this study were to determine whether CCH practice can enhance physical and mental health in elderly patients with mild cognitive impairment. The results indicated a slowed pace of memory decline. CCH is cost-effective and easy to practice. The training can be introduced by trainers who can assist older individuals by modifying their positions and focusing on their breathing, so they can perform CCH in daily life. Nonpharmacological therapies, such as CCH, etc., provide assistance to medications and can cultivate interest and develop physical and mental well-being, which are worth promoting. 

## 2. Materials and Methods 

It is easiest to grasp the order and location of strokes. To maximize the participants’ understanding of the line position and thickness of calligraphy characters and reduce the distraction of anticipating the next stroke, this study adopted the depicted writing method. The “Depict-Red” is a method of Chinese calligraphy in which practitioners place a copybook with red words under a thin sheet of paper and trace the characters. The participants trace and write in a regular font. The writers must read the Chinese characters first and think about the font and plan the stroke order before they begin to write [11,12] to allow the participants to see the position and thickness of the calligraphy model characters to the greatest extent and to reduce the distraction of thinking about the position of the pen.

**Figure 1:**
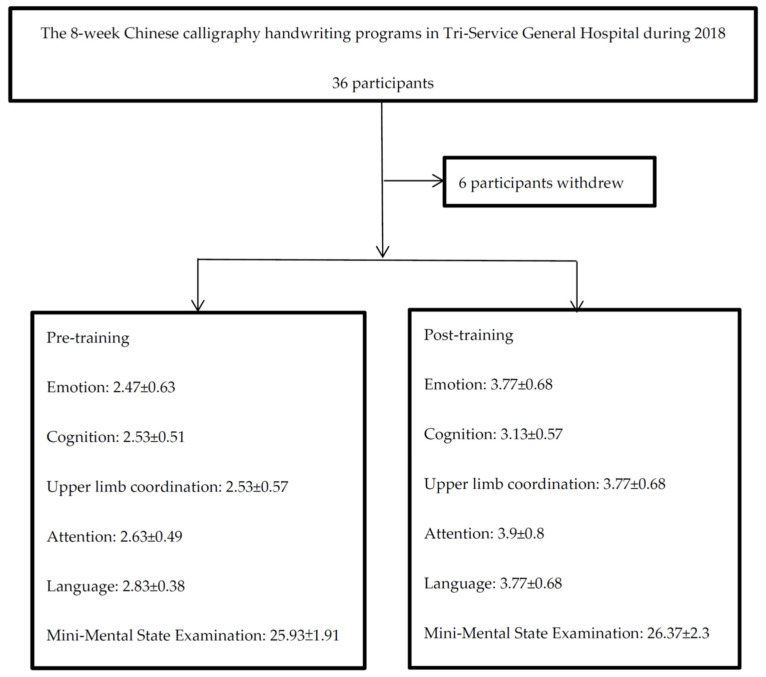
Flowchart of this study.

The three CCH programs in this study were conducted for 8 weeks from 11 April 2018 to 30 May 2018; 22 July 2018 to 17 August 2018; and 10 September 2018 to 2 November 2018. The programs were paid for with Taipei city funds designated for improving the quality of life of dementia patients and were free of charge to participants. The participants included memory decline patients identified by the Eight-item Informant Interview to Differentiate Aging and Dementia (AD8^®^), which helps discriminate between signs of normal aging and mild dementia by testing memory, orientation, judgment, and function [13], and their caregivers who were recruited by doctors, nurses, and cognitive function checkers. Mild cognitive impairment was diagnosed by Dr. Lin according to the diagnostic criteria of the Diagnostic and Statistical Manual of Mental Disorders, fifth edition, including symptoms of a significant decline in cognitive function and a substantial impairment in cognitive performance, no interference with capacity for independence in everyday activities, no delirium, and not better explained by another mental disorder [14]. Every participant initially received an introduction to the program and demonstration by Dr. Lin, participated in the CCH training for 8 weeks, and discussed their feelings and suggestions at the end of the program. The practice was performed in the discussion room at the Tri-Service General Hospital. There were 36 participants initially, but 6 withdrew due to personal reasons and were excluded from the final analysis. The intervention lasted 8 weeks, with two 1 h sessions each week. Tracing a copybook emphasizes practice, not the beauty and ugliness of the words, and there was no need to experience stress when practicing. The speed was slow to allow for concentrating instead of being distracted by other things. The participants shared their work through presentations during an open discussion on the last day of the course, which promoted language expression and a sense of accomplishment. Our study received ethical approval by TSGHIRB-1-107-05-187. A flowchart is shown in Figure 1.

We assessed participants’ psychological state, mental conditions, and motor skills with a questionnaire in the first session (first week) and the last session (eighth week) of the CCH program. The responses to the questions were measured on a 5-point scale: 1 indicated significant impairment; 2 indicated mild impairment or mild worsening; 3 indicated no effect or no impairment; 4 indicated mild improvements; and 5 indicated significant improvement. Using the stress response inventory [15], participants were assessed on a scale of 1 to 5 [15]: 1: frequent depression or anxiety; 2: sometimes depression or anxiety; 3: peaceful; 4: happy; or 5: satisfied. The participants’ memory and recall about persons, things, time, places, and objects were also assessed on a 5-point scale: 1: always forgetfulness (person they just met); 2: frequently forgetfulness (things, time, places, or objects); 3: good recall (with some impairment evident in complex affairs); 4: good performance (able to use tools); or 5: barrier free (able to perform activities of daily living). Upper limb function was assessed on a modified Fugl–Meyer Motor Scale [16]: 1: flexion and extension, free movement; 2: grasps paper well; 3: grasps pen well; 4: grasps bottle well with antigravity; or 5: grasps and throws ball well. Attention was assessed on a 5-point scale: 1: easily distracted; 2: sometimes distracted; 3: focused; 4: easily concentrated; or 5: excellent concentration. Language was also assessed on a 5-point scale: 1: communication barriers; 2: impaired fluency or comprehension; 3: fluency and comprehension intact without communication defects; 4: fluency and comprehension following common sense communication; or 5: logical communication (explaining one’s own work and correcting others’ shortcomings). 

A priori power analysis was performed to estimate the sample size. A large effect size has been reported. Therefore, we chose to use an effect size of d = 0.58 by referring to the suggestions of previous studies of older adults with mild cognitive impairment. With an alpha = 0.05 and power = 0.80, the total sample size was N = 26 [17].

Scores on the pretraining and post-training questionnaires and the Mini-Mental State Examination were analyzed with the nonparametric Wilcoxon signed-rank test. The changes by mean scores in emotional, memory, and motor functions are displayed by radar graphs. The statistical analysis was conducted using SPSS version 21 (Asia Analytics Taiwan Ltd., Taipei, Taiwan). 

## 3. Results

**Figure 2:**
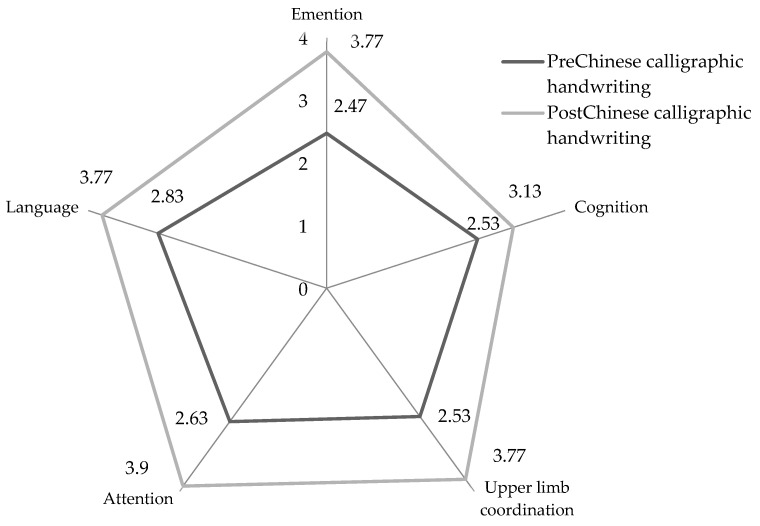
Radar chart showing the mean scores of emotional, memory, and motor functions before and after Chinese calligraphy handwriting training.

There were 7 males and 23 females who completed the 8 weeks of CCH training. The mean age was 72.07 ± 10.2 years old. Regarding the time of memory decline by patients’ and families’ statements, the time of memory impairment decline ranged from 0.5 to 1.5 years and the mean time of the disease was 0.82 ± 0.31 years (Table 1). We found a significant improvement in cognition, attention, emotion regulation, upper limb coordination, language, and the Mini-Mental State Examination scores (Table 2). The visual radar chart in Figure 2 shows the change after CCH training.

## 4. Discussion

This study found augmented cognitive, emotional, and upper limb stability in a self-report survey after CCH training in individuals with mild cognitive impairment. The CCH training involved a slowly writing pace and meditative breathing, resulting in higher concentration. Several practice sessions led the participants to adopt a slower pace of life and connected visuospatial and working memory about the form, sound, and meaning of Chinese characters as participants held a brush pen while maintaining upper limb stability. Language function was also improved after the oral presentations that participants gave while showing their work, similar to practices in art therapy. It is important to slow down rapid memory decline, maintain emotional stability, and create good times in patients with mild cognitive impairment through CCH training [6,7,8,9,10].

For many elderly individuals, CCH is an important memory from their childhood and adolescent learning during early school life in Taiwan. The CCH training course provided a wealth of sensory stimulation and opportunities for self-expression with open discussion and final presentations. During the course, the participants assisted each other, formed good interpersonal interactions, and increased interpersonal satisfaction, which increased their motivation and slowed functional decline.

A meta-analysis found a significant increase in cognition after CCH training [10]. Self-reported cognitive function in the domains of visuospatial memory and working memory showed significant improvements in the participants after CCH training in our study. CCH training involves visually reading and adjusting the words’ form, sound, and meaning and then writing the words by hand. The process stimulated the occipital, parietal, frontal cortex, and limbic system. Our study also found that Mini-Mental State Examination scores showed an increasing trend after CCH training. 

Elderly individuals can feel a sense of existence after they are retired, and the empty nest phenomenon of children leaving home may increase their anxiety and frustration. Patients with memory decline may experience changes in personality, coldness, and irritability. The participants obtained good interpersonal relationships, established friendships, induced positive thinking, and encouraged social willingness by helping each other through rewarding actions in the CCH course. CCH training was performed through breathing control that can modulate parasympathetic nerves to stabilize mental states. Through calligraphy training with other participants, older individuals can cultivate new interests and their anxiety can be alleviated. CCH training has been shown to increase alpha wave patterns and decrease heart rate and blood pressure. States of anxiety and distress were shown to be reduced after CCH training in type 2 diabetes mellitus patients. Cortical excitation assessed by theta rhythms from changes in the body, emotion, and cognition were significantly increased in the CCH group [18]. The midline of the brain and forehead usually have more and long-lasting low-frequency waves (1–3 Hz delta waves, 4–7 Hz theta waves) and fewer high-frequency waves (13–21 Hz beta waves), which is regarded as evidence of the low activation levels of the cerebral cortex [19]. CCH has been shown to relieve anxiety and depression in Chinese patients with nasopharyngeal carcinoma during the post-treatment period. CCH training in patients with cancer, similar to relaxation training, could slow physiological activity, increase concentration, and improve mood. After CCH training, improvements in insomnia were also noted [20]. Our study showed similar results for improvement in emotion regulation with persistent happiness and looking forward to practicing time.

Before writing, the participants need to observe the shape and stroke order of the Chinese charters with higher concentration. Physiological activity slowed with increasing concentration. A previous study found significant improvements in orientation, attention, and calculation in the CCH group compared with the control group, which showed a decline in orientation [21]. CCH training improved attention and communication activities in children with attention deficit hyperactivity disorder [22]. Small gray matter volume in the right precuneus/posterior cingulate cortex after long-term CCH training has been related to selective and divided attention. The treatment effects on augmenting working memory and verbal delayed memory were strong [23]. The rehearsal of the hang-style stroke before motor execution challenged working memory and was evidenced by increased digit span task (DST)-backward sequence scores. Two event-related potential components, N200 and P300, appear to be closely associated with the cognitive processes of perception and selective attention [24]. P300 latency has also been clinically applied as a diagnostic tool and a prognostic marker for recovery after cortical insult. Furthermore, the magnitude of alteration in P300 in the subacute phase of stroke correlated with functional recovery after several months. These behavioral changes were supported by event-related potentials with a longer N200 latency, modulation of encoding, and manipulation and shorter P300 latency with updating processes of the working memory content. A past study noted that CCH benefitted attention and working memory [25]. Our study showed similar results with more focused attention after CCH. 

The act of writing drives the functional adjustment of the limbs and body. While writing Chinese calligraphy starts with holding a brush pen with soft and flexible bristles, the hand needs better stability and intervening connections of the basal ganglion and cerebellar neurons in the brain. In the process of writing, lifting, and pressing with different strengths will result in strokes showing differences in thickness, which can train subtle coordination. A previous study revealed that CCH could affect patients’ motor activity with a positioning task and task time. A previous study found improvements in palm motor function and coordination with 2 weeks of CCH training in stroke patients. But the results of electromyography studies were insignificant, although there was an increasing trend [5]; longer training may have a significant effect. The participants opened the ink tank, dipped the brush in ink, and started to trace the characters to write during every practice session with increased hand muscle activity. The patients needed to hold a brush pen and write words during every training session, to train and strengthen their palm muscles and arms. These actions are executed through the frontal motor cortex and handwriting activates the muscles modulated by the basal ganglion and cerebellum [26]. A previous study used audiovisual integrative training to improve cognition and motor functions [27]. After 8 weeks of CCH training, the participants felt significantly better about upper limb coordination compared with the pretraining condition in this study.

The geometric qualities and textual implications of Chinese characters during CCH training involve the process of cognitive formation allowing the writers to obtain cognitive stimulation. These handwriting exercises are used to learn to read and draw Chinese characters. There were improvements in writing speed and accuracy, abstract thinking, and spatial ability after CCH practice. Cortical activities and arousal were improved after CCH training compared to baseline [28]. Past studies have suggested that the visual-spatial attributes of Chinese characters can promote the spelling process of characters, as CCH effectively activates the right brain. The superior medial frontal cortex plays a role in inhibitory control related to CCH skills. The thalamus plays an important role in new learning, inhibitory control, and motor control associated with the integration that occurs in CCH training. The cingulate cortex plays an important role in emotional processes. Smaller volumes of gray matter in the precuneus/posterior cingulate cortex are deactivated while meditating with a focus on attention [29]. The CCH group was shown to have greater resting-state functional connectivity with better executive functions involving the frontal, parietal, and basal ganglia than the control group with long-term handwriting training [30]. The increased theta waves associated with working memory improvements involved the dorsolateral and superior frontal cortex. CCH training occurs through visual information input to recognize the structure, sound, and meaning by the thalamus sensation, then pass the signal to the frontal executive area with basal ganglion coordination. The sensory speech area is located in Wernicke’s area and the motor speech area is located in Broca’s area connected with conduction fibers [31]. At the end of the course, the participants explored their galleries and discussed and shared their feelings with each other to promote language expression and a sense of achievement, which increased their self-confidence, self-esteem, and organization skills. Speech function seemed to improve after CCH training in our study through Chinese word learning and speaking, indicating enhanced functions of the supratemporal lobe and inferior frontal lobe, which has been less explored in past studies.

The physiological changes resulting from CCH training through qi’s operation include slower respiration and heart rate and reduced blood pressure with enhanced parasympathetic function. Hyperarousal symptoms and salivary cortisol levels were reduced after CCH therapy in child survivors suffering from earthquakes with hyper-sympathetic modulation [32]. A brain imaging study comparing a long-term CCH group with a control group showed effects in the limbic system [29]. CCH seems to have modified autonomic function in past studies. A one-year study of patients with Alzheimer’s disease in nursing homes showed that one-hour exercises conducted twice every week, including physical fitness courses for walking, physical fitness, balance, and physical flexibility, can attenuate patients’ daily activity decline [33]. Although the heart rate variability (HRV) study did not show significant parasympathetic reversal in dementia patients, the potential reason was that the duration of CCH was too short to show a difference [34]. Through additional research with music, the effect of calligraphy writing between Guqin music sessions showed increasing HRV coherence [23]. 

Nonpharmacological therapies in elderly populations can use familiar activities based on their past, so that they are willing to be contacted and participate in the activity again. The CCH training could achieve qi calming divine condensation. The participants relearned characters, practiced CCH, then shared their opinions and interpretations in the course. CCH can be used as a medium to encourage elderly individuals with mild cognitive impairment to achieve the effects of concentration, enjoyment, safety, happiness, and stability. Our study performed a self-reported assessment of functional recovery following CCH training to understand its effect on older individuals. We used semantic visualization of the changes in emotional state, movement, and memory after older people persistently practiced CCH every week for 2 months. The initial beneficial effect of medications followed long-term disease progression [35]. In the face of dementia that cannot be cured with medications [4], prevention of memory decline with multiple nonpharmacological interventions is the clear direction. CCH enhanced the participants’ health by slowing the pace of life and modifying their deep thinking. It can help elderly people relax their minds, improve their physical, mental, and spiritual health, and achieve healthcare effectiveness.

There are some limitations in this study. First, this study lacked a control group to assess the absolute benefit of CCH training. A comparison study needs to be performed in the future. Second, this study assessed patients’ subjective self-reporting; objective exams were unavailable and further study needs to be designed with professional tests. Because the CCH program was provided with free of charge due to government support, the effect of CCH may be overestimated. Third, there were more female patients involved as a result of their higher interest in the new practices. Future designs should provide more of a balance in the sexes of participants. Females have a higher motivation to learn and engage in social activity, and males need be encouraged to participate in programs that could benefit those in need of chronic care. Fourth, the Mini-Mental State Examination scores showed rank-sum improvement rather than variance analysis. The potential reason is that older participants had milder cognitive decline before CCH training; thus, posttraining mild functional recovery did not reach a significant difference in the analysis of variance. In addition, the Mini-Mental State Examination scores needed a sample size of N = 55; thus, more subjects need to be enrolled and evaluated using this score in the future [36]. Fifth, although there was a good effect of short-term CCH training, the effect of long-term CCH training for mild cognitive impairment was not available in this study. A past study found that exercise intervention over a one-year period did not change cognitive performance in patients with mild cognitive impairment compared to a non-exercise group [37]. The long-term CCH practice had a more mature Confucian literati personality in a past study [8]. Prospective studies are needed to evaluate the long-term effects in the future. 

## 5. Conclusions

This study found self-reported functional improvements in a mild memory decline after CCH training. The participants’ psychological symptoms improved. Improvements in the ability to focus, hand steadiness, and language fluency were observed. Short-term CCH training for patients with mild cognitive impairment could improve concentration, emotional regulation, muscle strength stability, and self-awareness. Long-term care units could use the evidence from this study to support CCH programs for patients with memory decline with significant benefits.

## Figures and Tables

**Table 1:** Baseline characteristics of the study participants.

Variables	
Sex	7 males and 23 females
Age	72.07 ± 10.2
Education years (range)	14.07 ± 2.94 (6–18)
Years of the disease (range)	0.82 ± 0.31 (0.5–1.5)
Mini-Mental State Examination score	25.93 ± 1.91

**Table 2:** Functional changes before and after Chinese calligraphy handwriting training.

	PreCCH	PostCCH	*p*
Emotion	2.47 ± 0.63	3.77 ± 0.68	<0.001 *
Cognition	2.53 ± 0.51	3.13 ± 0.57	<0.001 *
Upper limb coordination	2.53 ± 0.57	3.77 ± 0.68	<0.001 *
Attention	2.63 ± 0.49	3.9 ± 0.8	<0.001 *
Language	2.83 ± 0.38	3.77 ± 0.68	<0.001 *
Mini-Mental State Examination	25.93 ± 1.91	26.37 ± 2.3	0.008 *

Wilcoxon sign test for nonparametric variables. CCH: Chinese calligraphy handwriting. * *p* < 0.05.

## Data Availability

The datasets used in the current study are available from the corresponding author upon reasonable request.

## References

[1] Lanctôt K.L., Amatniek J., Ancoli-Israel S., Arnold S.E., Ballard C., Cohen-Mansfield J., Ismail Z., Lyketsos C., Miller D.S., Musiek E. (2017). Neuropsychiatric signs and symptoms of Alzheimer’s disease: New treatment paradigms. Alzheimers Dement. Transl. Res. Clin. Interv..

[2] World Health Organization (2018). Dementia: Number of People Affected to Triple in Next 30 Years. https://www.who.int/news/item/07-12-2017-dementia-number-of-people-affected-to-triple-in-next-30-years.

[3] Qiu C., Kivipelto M., von Strauss E. (2009). Epidemiology of Alzheimer’s disease: Occurrence, determinants, and strategies toward intervention. Dialogues Clin. Neurosci..

[4] Lynch C. (2020). World Alzheimer Report 2019: Attitudes to dementia, a global survey: Public health: Engaging people in ADRD research. Alzheimer’s Dement..

[5] Lee N.R., Park Y.J., Jang J.S. (2021). Analysis of Effect of Non-drug intervention on the Elderly with Dementia in Korea: Meta-Analysis. J. Korea Acad.-Ind. Coop. Soc..

[6] Kao H.S. (2006). Shufa: Chinese calligraphic handwriting (CCH) for health and behavioural therapy. Int. J. Psychol..

[7] Kao H.S. (2010). Calligraphy therapy: A complementary approach to psychotherapy. Asia Pac. J. Couns. Psychother..

[8] Kao H.S., Xu M., Kao T.T. (2021). Calligraphy, psychology and the Confucian literati personality. Psychol. Dev. Soc..

[9] Kao H.S., Zhu L., Chao A.A., Chen H.Y., Liu I.C., Zhang M. (2014). Calligraphy and meditation for stress reduction: An experimental comparison. Psychol. Res. Behav. Manag..

[10] Chu K.-Y., Huang C.-Y., Ouyang W.-C. (2018). Does Chinese calligraphy therapy reduce neuropsychiatric symptoms: A systematic review and meta-analysis. BMC Psychiatry.

[11] Wu Y., Yuan Z., Zhou D., Cai Y. Research of virtual Chinese calligraphic learning. Proceedings of the 2013 IEEE International Conference on Multimedia and Expo (ICME).

[12] Liang C.W., Hsieh M.C., Young K.Y. (2011). A VR-based calligraphy writing system with force reflection. Int. J. Autom. Smart Technol..

[13] Galvin J.E., Roe C.M., Powlishta K.K., Coats M.A., Muich S.J., Grant E., Miller J.P., Storandt M., Morris J.C. (2005). The AD8: A brief informant interview to detect dementia. Neurology.

[14] American Psychiatric Association (2013). Diagnostic and Statistical Manual of Mental Disorders.

[15] Koh K.B., Park J.K., Kim C.H., Cho S. (2001). Development of the stress response inventory and its application in clinical practice. Psychosom. Med..

[16] Hsieh Y.W., Hsueh I.P., Chou Y.T., Sheu C.F., Hsieh C.L., Kwakkel G. (2007). Development and validation of a short form of the Fugl-Meyer motor scale in patients with stroke. Stroke.

[17] Wong W.P., Coles J., Chambers R., Wu D.B.C., Hassed C. (2017). The effects of mindfulness on older adults with mild cognitive impairment. J. Alzheimer’s Dis. Rep..

[18] Xu M., Kao H.S., Zhang M., Lam S.P., Wang W. (2013). Cognitive-neural effects of brush writing of Chinese characters: Cortical excitation of theta rhythm. Evid.-Based Complement. Altern. Med..

[19] Monastra V.J., Monastra D.M., George S. (2002). The effects of stimulant therapy, EEG biofeedback, and parenting style on the primary symptoms of attention-deficit/hyperactivity disorder. Appl. Psychophysiol. Biofeedback.

[20] Wagner S. (2018). Calligraphy therapy interventions for managing depression in cancer patients: A scoping study. Altern. Integ. Med..

[21] Kwok T.C., Bai X., Kao H.S., Li J.C., Ho F.K. (2011). Cognitive effects of calligraphy therapy for older people: A randomized controlled trial in Hong Kong. Clin. Interv. Aging.

[22] Shen I.-H., Lee T.-Y., Chen C.-L. (2012). Handwriting performance and underlying factors in children with Attention Deficit Hyperactivity Disorder. Res. Dev. Disabil..

[23] Chan C.C., Derbie A.Y., Hui I., Pang M.Y.C., Fong K.N., Chan S.C. (2018). Chinese calligraphic writing to enhance cognitive performance and emotional calmness in older adults with mild cognitive impairment. Hong Kong Med. J..

[24] Patel S.H., Azzam P.N. (2005). Characterization of N200 and P300: Selected studies of the event-related potential. Int. J. Med. Sci..

[25] Chan S.C.C., Chan C.C.H., Derbie A.Y., Hui I., Tan D.G.H., Pang M.Y.C., Lau S.C.L., Fong K.N.K. (2017). Chinese Calligraphy Writing for Augmenting Attentional Control and Working Memory of Older Adults at Risk of Mild Cognitive Impairment: A Randomized Controlled Trial. J. Alzheimers Dis..

[26] Svoboda K., Li N. (2018). Neural mechanisms of movement planning: Motor cortex and beyond. Curr. Opin. Neurobiol..

[27] Lee L.P., Har A.W., Ngai C.H., Lai D.W.L., Lam B.Y., Chan C.C. (2020). Audiovisual integrative training for augmenting cognitive- motor functions in older adults with mild cognitive impairment. BMC Geriatr..

[28] Kao H.S., Ding-Guo G., Danmin M., Xufeng L. (2004). Cognitive facilitation associated with Chinese brush handwriting: The case of symmetric and asymmetric Chinese characters. Percept. Mot. Ski..

[29] Chen W., Chen C., Yang P., Bi S., Liu J., Xia M., Lin Q., Ma N., Li N., He Y. (2019). Long-term Chinese calligraphic handwriting reshapes the posterior cingulate cortex: A VBM study. PLoS ONE.

[30] Chen W., He Y., Gao Y., Zhang C., Chen C., Bi S., Yang P., Wang Y., Wang W. (2017). Long-term experience of Chinese calligraphic handwriting is associated with better executive functions and stronger resting-state functional connectivity in related brain regions. PLoS ONE.

[31] Nadeau S.E. (2021). Language and Aphasias. Continuum Minneap. Minn..

[32] Zhu Z., Wang R., Kao H.S., Zong Y., Liu Z., Tang S., Xu M., Liu I.C., Lam S.P. (2014). Effect of calligraphy training on hyperarousal symptoms for childhood survivors of the 2008 China earthquakes. Neuropsychiatr. Dis. Treat..

[33] Telenius E.W., Engedal K., Bergland A. (2015). Effect of a high-intensity exercise program on physical function and mental health in nursing home residents with dementia: An assessor blinded randomized controlled trial. PLoS ONE.

[34] Lam S.P., Kao H.S., Kao X., Fung M.M.Y., Kao T.T. (2019). HRV Regulation by Calligraphic Finger-writing and Guqin Music: A Pilot Case Study. NeuroRegulation.

[35] Birks J. (2006). Cholinesterase inhibitors for Alzheimer’s disease. Cochrane Database Syst. Rev..

[36] Cohen J. (1988). Statistical Power Analysis for the Behavioral Sciences.

[37] Stuckenschneider T., Sanders M.L., Devenney K.E., Aaronson J.A., Abeln V., Claassen J.A.H.R., Guinan E., Lawlor B., Meeusen R., Montag C. (2021). NeuroExercise: The Effect of a 12-Month Exercise Intervention on Cognition in Mild Cognitive Impairment-A Multicenter Randomized Controlled Trial. Front. Aging Neurosci..

